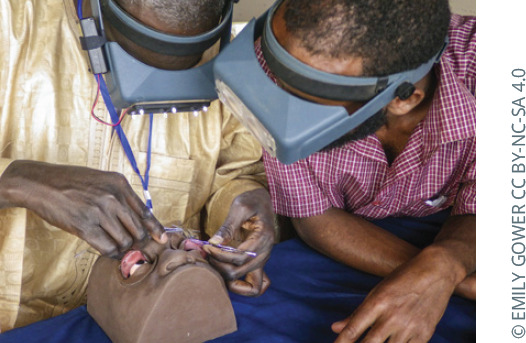# Learning surgical skills for eye care

**Published:** 2023-12-01

**Authors:** William Dean, Daksha Patel, Rengaraj Venkatesh, Elmien Wolvaardt

**Affiliations:** 1 Assistant Clinical Professor: ICEH, LSHTM, London, UK.; 2 Associate Professor, ICEH, LSHTM, London, UK.; 3 Chief Medical Officer: Aravind Eye Hospital, Pondicherry, India.; 4 Editor-in-Chief: *Community Eye Health Journal*, ICEH, LSHTM, London, UK.

**Advances in training methods and technology allow surgical teams to engage in sustained and deliberate practice, initially safely away from patients.**

**Figure:**
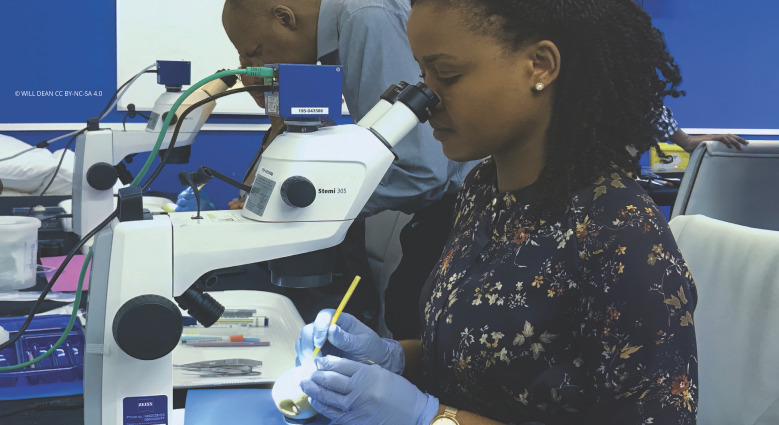
Practicing making a scleral tunnel, using an apple in a digital dry lab. **SOUTH AFRICA** © WILL DEAN CC BY-NC-SA 4.0

Surgery plays a vital role in global eye care by addressing a wide range of eye conditions, from cataract to more complex eye diseases and injuries.

Our aim with this issue is to support the training and development of eye surgeons and surgical team members by sharing innovative, impactful, and proven ways to learn, practice, and teach surgical and technical skills.

Surgery used to be learnt only on patients, in accordance with the traditional approach of “see one, do one, teach one.”^1^ Advances in surgical simulation and the development of surgical competency rubrics have made it possible for surgeons to learn and practice in a systematic way, before operating on patients.

Competency assessment rubrics, such as the Ophthalmology Surgical Competency Assessment Rubrics (OSCARs),^2^ divide surgical procedures into individual steps, each with four clearly defined grades: Novice, Beginner, Advanced Beginner, and Competent. Ophthalmic simulated surgical competency assessment rubrics (Sim-OSSCARs) have also been developed and validated for learning and practising surgical steps in simulation.^3,4^

The steps in competency assessment rubrics are demonstrated or taught one at a time. Trainees then practice each step, with appropriate supervision and feedback, until they are graded ‘Competent.’

Another key building block for developing and enhancing surgical skills is **reflective learning**. Reflective learning is a continuous cycle that enables individuals to improve by critically evaluating their own performance against the criteria set in the relevant OSCAR or Sim-OSSCAR. It follows a cycle of practice, observation, error detection, trying a change in technique, and observing any differences in outcome.

## Simulation training

Sustained deliberate practice – with or without feedback from mentors or trainers – is only possible thanks to the increasing availability of simulation training, where skills can be practiced away from patients.

Simulation training offers the surgeon an accessible, safe, and reproducible method of learning. Simulation training, also known as simulation-based surgical education, can be divided into four main types, in order of increasing cost:

1. Practice on foam sheets or fruit, with or without microscopes and microsurgical instruments
2. Practice on animal or human cadaver eyes (in a wet lab)
3. High-fidelity simulation, using artificial eyes.
4. Virtual reality (VR) simulation systems.

Simulation allows trainees to make mistakes and reflect without risking patient safety. Technological advances in virtual reality simulation can also provide practice in the management of complications, and surgical teams can practice handling these together.

Advanced simulation technology may be a costly initial costly investment, and it is always worth exploring low-cost options to support training.^5^

## The surgical team

In this issue, we also provide tips and guidance for scrub nurses/technicians. By practising together in a simulated surgical set-up, nurses and surgeons can develop the communication and manual skills needed to improve surgical outcomes and protect patients – which includes practising the World Health Organization Safe Surgery check-in and check-out procedures.

All of this is not to say that the old adage of “see one, do one, teach one” has no value – far from it. Once surgeons and their teams have made the most of simulated training opportunities, they will gain vital – and essential – experience by observing experienced colleagues and practising under their supervision, provided that the patients they operate on have been carefully selected to ensure the operation is both safe for the patient, and a valuable learning opportunity for the surgeon and the surgical team.

**Read more online!**

**Available at www.cehjournal.org and our app: www.cehjournal.org**

**Figure:**
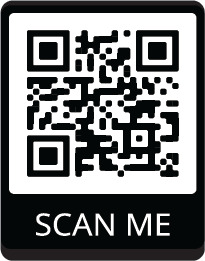

**Cybersight: improving remote access to surgical training and mentoring**

Maria Jose Montero, Hannah Marr, Nathan Congdon, Meryem Altun and Alana Calise

Remote mentoring can provide affordable access to surgical training, even in low-resource settings.

**Figure:**
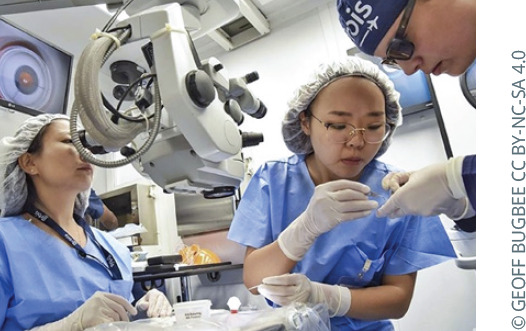

**More than simulation: the HelpMeSee approach to cataract surgical training**

Van Charles Lansingh and Akshay Gopinathan Nair

Despite the many benefits of virtual reality surgical simulation, trainees still benefit from pre-learning and the presence of experienced instructors.

**Figure:**
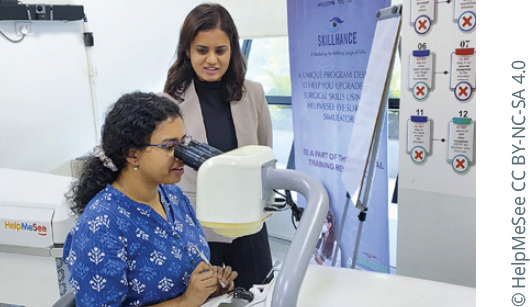

**Wet lab and live surgical training at Aravind Hospitals**

Sankarananthan R, Senthil Prasad R, Dhivya Ramasamy, Thulasiraj D Ravilla and Madhu Shekhar

Wet lab and live surgical training are both vital components of the residency programme in ophthalmology at Aravind Eye Hospitals in India.

**Figure:**
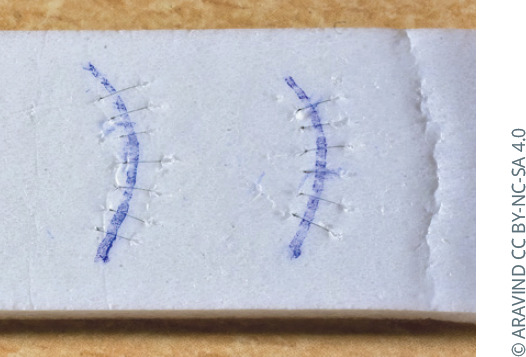

**Low-cost remote wet lab training in corneal surgery in India: LV Prasad Eye Institute**

Kavya Chandran, Karthikesh Anche, Pravin Vaddavalli and Padmaja Kumari Rani

A low-cost remote wet lab model developed during the COVID-19 pandemic continues to be useful, by eliminating the need for surgical fellows to travel long distances.

**Figure:**
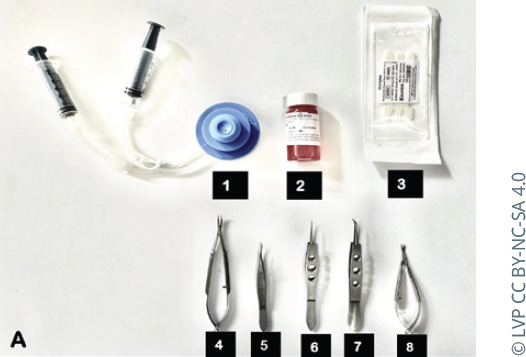

**Maintaining high quality trichiasis surgery before and after trachoma elimination**

Emily Gower, Belay Bayissasse, Amir B Kello and Tim Jesudason

Surgical simulation training can help to maintain the quality of trichiasis surgery in a post-elimination setting.

**Figure:**